# Supplementary material for: A UMLS-based spell checker for natural language processing in vaccine safety
Source: BMC Med Inform Decis Mak. 2007 Feb 12;7:3. doi: 10.1186/1472-6947-7-3 (PMC1805499; doi:10.1186/1472-6947-7-3)
Supplement: Additional file 13 — RAP application directory. Sets up directory for RAP (RDF application for PHP) source code [file 1472-6947-7-3-S13.gz › rap/doc/phpdoc/__filesource/fsource_ontModel__ontModelOntResource.php.html]

xml version="1.0" encoding="iso-8859-1"?


File Source for OntResource.php


# Source for file OntResource.php

Documentation is available at OntResource.php

```
1. <?php
2. /**
3. * ----------------------------------------------------------------------------------
4. * Class: OntResource
5. * ----------------------------------------------------------------------------------
6. *
7. * @package     ontModel
8. */


11. /**
12. * Provides a common super-type for all of the abstractions in
13. * this ontology representation package.
14. *
15. * <BR><BR>History:
16. * <LI>10-05-2004                : First version of this class.</LI>
17. *
18. * @version  V0.9.1
19. * @author Daniel Westphal <mail at d-westphal dot de>
20. *
21. *
22. * @package     ontModel
23. * @access    public
24. ***/
25. class OntResource extends ResResource
26. {
27. /**
28. * Holds a reference to the assoiated vocabulary
29. * @var        object
30. * @access    private
31. */
32. var $vocabulary;

34. /**
35. * Holds a resResource of the type, which is this ontResource of.
36. * If this value is set, the ontModel will add an additional
37. * statement about this resource and the fiven rdf:type
38. * @var        object
39. * @access    private
40. */
41. var $rdfType;


44. /**
45. * Constructor
46. * You can supply a uri
47. *
48. * @param string $uri
49. * @access    public
50. */
51. function OntResource($uri = null)
52. {
53. $this->rdfType=false;
54. parent::ResResource($uri);
55. }

57. /**
58. * Sets the reference to the assoiated vocabulary
59. *
60. * @param    object OntVocabulary    $vocabulary
61. * @access    public
62. */
63. function setVocabulary(& $vocabulary)
64. {
65. $this->vocabulary = & $vocabulary;
66. }

68. /**
69. * Add the given comment to this resource.
70. *
71. * @param    object ResLiteral    $comment
72. * @return    boolean
73. * @access    public
74. */
75. function addComment($comment)
76. {
77. return $this->addProperty($this->vocabulary->COMMENT(),$comment);
78. }

80. /**
81. * Answer the comment string for this object. If there is more than one such resource, an arbitrary selection is made.
82. *
83. * @return    object ResLiteral or NULL
84. * @access    public
85. */
86. function getComment()
87. {
88. return $this->getPropertyValue($this->vocabulary->COMMENT());
89. }

91. /**
92. * Add a resource that is declared to provide a definition of this resource.
93. *
94. * @param    object ResResource    $resResource
95. * @return    boolean
96. * @access    public
97. */
98. function addIsDefinedBy($resResource)
99. {
100. return $this->addProperty($this->vocabulary->IS_DEFINED_BY(),$resResource);
101. }

103. /**
104. * Answer a resource that is declared to provide a definition of this resource.
105. * If there is more than one such resource, an arbitrary selection is made.
106. *
107. * @return    object ResResource
108. * @access    public
109. */
110. function getIsDefinedBy()
111. {
112. return $this->getPropertyValue($this->vocabulary->IS_DEFINED_BY());
113. }

115. /**
116. * Add the given Label to this resource
117. *
118. * @param    object ResLiteral    $resLiteral
119. * @return    boolean
120. * @access    public
121. */
122. function addLabelProperty($resLiteral)
123. {
124. return $this->addProperty($this->vocabulary->LABEL(),$resLiteral);
125. }

127. /**
128. * Answer the label ResLiteral for this object.
129. * If there is more than one such resource, an arbitrary selection is made.
130. *
131. * @param    string    $uri
132. * @return    object ResResource
133. * @access    public
134. */
135. function getLabelProperty()
136. {
137. return $this->getPropertyValue($this->vocabulary->LABEL());
138. }

140. /**
141. * Add the given class as one of the rdf:type's for this resource.
142. *
143. * @param    object ResResource    $resResource
144. * @return    boolean
145. * @access    public
146. */
147. function addRDFType($resResource)
148. {
149. return $this->addProperty($this->vocabulary->TYPE(),$resResource);
150. }

152. /**
153. * Answer the rdf:type (ie the class) of this resource.
154. * If there is more than one type for this resource, the return value will
155. * be one of the values, but it is not specified which one
156. * (nor that it will consistently be the same one each time).
157. *
158. * @return    object ResResource
159. * @access    public
160. */
161. function getRDFType()
162. {
163. return $this->getPropertyValue($this->vocabulary->TYPE());
164. }

166. /**
167. * Add a resource that is declared to provided additional
168. * information about the definition of this resource.
169. *
170. * @param    object ResResource    $resResource
171. * @return    boolean
172. * @access    public
173. */
174. function addSeeAlso($resResource)
175. {
176. return $this->addProperty($this->vocabulary->SEE_ALSO(),$resResource);
177. }

179. /**
180. * Answer a resource that provides additional information about this resource.
181. * If more than one such resource is defined, make an arbitrary choice.
182. *
183. * @return    object ResResource
184. * @access    public
185. */
186. function getSeeAlso()
187. {
188. return $this->getPropertyValue($this->vocabulary->SEE_ALSO());
189. }

191. /**
192. * Answer a view of this resource as a class
193. *
194. * @return    object OntClass
195. * @access    public
196. */
197. function asClass()
198. {
199. return  $this->model->createOntClass($this->uri);
200. }

202. /**
203. * Answer a view of this resource as an Individual
204. *
205. * @return    object Individual
206. * @access    public
207. */
208. function asIndividual()
209. {
210. return  $this->model->createIndividual($this->uri);
211. }

213. /**
214. * Answer a view of this resource as a property
215. *
216. * @return    object OntProperty
217. * @access    public
218. */
219. function asOntProperty()
220. {
221. return  $this->model->createOntProperty($this->uri);
222. }

224. /**
225. * Answer a reference to the ontology language profile that governs the
226. * ontology model to which this ontology resource is attached.
227. *
228. * @param    string    $uri
229. * @return    object OntClass
230. * @access    public
231. */
232. function getVocabulary()
233. {
234. return $this->vocabulary ;
235. }

237. /**
238. * Answer the value of a given RDF property for this resource as $returnType, or null
239. * if it doesn't have one. If there is more than one RDF statement with
240. * the given property for the current value, it is not defined which of
241. * the values will be returned.
242. * The following return Types are supported: 'OntClass', 'OntProperty', 'Individual', and 'ResResource'
243. * Default is 'ResResource'
244. *
245. * @param    object ResResource    $property
246. * @param    string    $returnType
247. * @return    object OntClass
248. * @access    public
249. */
250. function getPropertyValue($property, $returnType = 'ResResource')
251. {
252. $statement=$this->getProperty($property);
253. if ($statement===null)
254. return null;

256. switch ($returnType)
257. {
258. case 'OntClass':
259. return $this->model->createOntClass($statement->getLabelObject());
260. break;

262. case 'OntProperty':
263. return $this->model->createOntProperty($statement->getLabelObject());
264. break;

266. case 'Individual':
267. return $this->model->createIndividual($statement->getLabelObject());
268. break;

270. default:
271. return $statement->getObject();
272. break;
273. }
274. }

276. /**
277. * Answer true if this resource has the given comment.
278. *
279. * @param    object ResLiteral    $resLiteral
280. * @return    boolean
281. * @access    public
282. */
283. function hasComment($resLiteral)
284. {
285. return $this->hasProperty($this->vocabulary->COMMENT(),$resLiteral);
286. }

288. /**
289. * Answer true if this resource has the given label.
290. *
291. * @param    object ResLiteral    $resLiteral
292. * @return    boolean
293. * @access    public
294. */
295. function hasLabelProperty($resLiteral)
296. {
297. return $this->hasProperty($this->vocabulary->LABEL(),$resLiteral);
298. }

300. /**
301. * Answer true if this resource has the given rdf:type.
302. *
303. * @param    object ResResource    $ontClass
304. * @return    boolean
305. * @access    public
306. */
307. function hasRDFType($ontClass)
308. {
309. return $this->hasProperty($this->vocabulary->TYPE(),$ontClass);
310. }

312. /**
313. * Answer true if this resource has the given resource as a source
314. * of additional information.
315. *
316. * @param    object ResResource    $resResource
317. * @return    boolean
318. * @access    public
319. */
320. function hasSeeAlso($resResource)
321. {
322. return $this->hasProperty($this->vocabulary->SEE_ALSO(),$resResource);
323. }

325. /**
326. * Answer true if this resource is defined by the given resource.
327. *
328. * @param    object ResResource    $resResource
329. * @return    boolean
330. * @access    public
331. */
332. function isDefinedBy($resResource)
333. {
334. return $this->hasProperty($this->vocabulary->IS_DEFINED_BY(),$resResource);
335. }

337. /**
338. * Answer an array of all of the comment literals for this resource.
339. *
340. * @param    string    $language
341. * @return    array
342. * @access    public
343. */
344. function listComments($language)
345. {
346. $return=$this->listProperty($this->vocabulary->COMMENT());
347. if ($language === false)
348. return $return;

350. foreach ($return as $key => $resLiteral)
351. {
352. if (!is_a($resLiteral,'ResLiteral') || $resLiteral->getLanguage() != $language)
353. unset ($return[$key]);
354. }
355. return $return;
356. }

358. /**
359. * Answer an array of all of the resources that are declared to define this resource.
360. *
361. * @return    array
362. * @access    public
363. */
364. function listIsDefinedBy()
365. {
366. return $this->listProperty($this->vocabulary->IS_DEFINED_BY());
367. }

369. /**
370. * Answer an array of all of the label literals for this resource, with the given
371. * language, if $language is set.
372. *
373. * @return    array
374. * @access    public
375. */
376. function listLabelProperties($language = false)
377. {
378. $return=$this->listProperty($this->vocabulary->LABEL());
379. if ($language === false)
380. return $return;

382. foreach ($return as $key => $resLiteral)
383. {
384. if (!is_a($resLiteral,'ResLiteral') || $resLiteral->getLanguage() != $language)
385. unset ($return[$key]);
386. }
387. return $return;

389. }

391. /**
392. * Answer an array of the RDF classes to which this resource belongs.
393. * If $direct is true, only answer those resources that are direct types of
394. * this resource, not the super-classes of the class etc.
395. *
396. * @param    boolean    $direct
397. * @return    array Array of ResResources
398. * @access    public
399. */
400. function listRDFTypes($direct = true)
401. {
402. return $this->listProperty($this->vocabulary->TYPE());
403. }

405. /**
406. * Answer an array of all of the resources that are declared to
407. * provide addition information about this resource.
408. *
409. * @return    array Array of ResResources
410. * @access    public
411. */
412. function listSeeAlso()
413. {
414. return $this->listProperty($this->vocabulary->SEE_ALSO());
415. }

417. /**
418. * Answer an array of values of a given RDF property for this resource as $returnType, or null
419. * if it doesn't have one.
420. * The following return Types are supported: 'OntClass', 'OntProperty', 'Individual', and 'ResResource'
421. * Default is 'ResResource'
422. *
423. * @param    object ResResource    $property
424. * @param    string    $returnType
425. * @return    array of ResResources
426. * @access    public
427. */
428. function listProperty($property, $returnType = 'OntResource')
429. {
430. $return=array();
431. $resArray = $this->listProperties($property);
432. foreach ($resArray as $statement)
433. {
434. switch ($returnType)
435. {
436. case 'OntClass':
437. $return[]=$this->model->createOntClass($statement->getLabelObject());
438. break;

440. case 'OntProperty':
441. $return[]=$this->model->createOntProperty($statement->getLabelObject());
442. break;

444. case 'Individual':
445. $return[]=$this->model->createIndividual($statement->getLabelObject());
446. break;

448. default:
449. $return[]=$statement->getObject();
450. break;
451. }
452. }
453. return $return;
454. }

456. /**
457. * Remove the statement that the given ResLiteral is a comment on this resource.
458. * Returns true, if a statement was removed
459. *
460. * @param    object ResLiteral $resLiteral
461. * @return    boolean
462. * @access    public
463. */
464. function removeComment($resLiteral)
465. {
466. return $this->removeProperty($this->vocabulary->COMMENT(),$resLiteral);
467. }

469. /**
470. * Remove the statement that this resource is defined by the given resource.
471. *
472. * @param    object ResResource $resResource
473. * @return    boolean
474. * @access    public
475. */
476. function removeDefinedBy($resResource)
477. {
478. return $this->removeProperty($this->vocabulary->IS_DEFINED_BY(),$resResource);
479. }

481. /**
482. * Remove the statement that the given ResLiteral is a label on this resource.
483. * Returns true, if a statement was removed
484. *
485. * @param    object ResLiteral $resLiteral
486. * @return    boolean
487. * @access    public
488. */
489. function removeLabelProperty($resLiteral)
490. {
491. return $this->removeProperty($this->vocabulary->LABEL(),$resLiteral);
492. }

494. /**
495. * Remove the specific property-value pair from this resource.
496. *
497. * @param    object ResResource    $property
498. * @param    object ResResource    $value
499. * @return    boolean
500. * @access    public
501. */
502. function removeProperty($property, $value)
503. {
504. return $this->model->remove(new Statement($this,$property,$value));
505. }

507. /**
508. * Remove the statement that this resource is of the given RDF type.
509. *
510. * @param    object ResResource $resResource
511. * @return    boolean
512. * @access    public
513. */
514. function removeRDFType($resResource)
515. {
516. return $this->removeProperty($this->vocabulary->TYPE(),$resResource);
517. }

519. /**
520. * Remove the statement indicating the given resource as a source of
521. * additional information about this resource.
522. *
523. * @param    object ResResource $resResource
524. * @return    boolean
525. * @access    public
526. */
527. function removeSeeAlso($resResource)
528. {
529. return $this->removeProperty($this->vocabulary->SEE_ALSO(),$resResource);
530. }

532. /**
533. * Assert that the given string is the comment on this resource.
534. * Any existing statements for comment will be removed.
535. *
536. * @param    object ResLiteral    $resLiteral
537. * @access    public
538. */
539. function setComment($resLiteral)
540. {
541. $this->setPropertyValue($this->vocabulary->COMMENT(),$resLiteral);
542. }

544. /**
545. * Assert that the given resource provides a source of definitions about this resource.
546. * Any existing statements for isDefinedBy will be removed.
547. *
548. * @param    object ResResource    $resResource
549. * @access    public
550. */
551. function setIsDefinedBy($resResource)
552. {
553. $this->setPropertyValue($this->vocabulary->IS_DEFINED_BY(),$resResource);
554. }

556. /**
557. * Assert that the given string is the label on this resource.
558. * Any existing statements for comment will be removed.
559. *
560. * @param    object ResLiteral    $resLiteral
561. * @access    public
562. */
563. function setLabelProperty($resLiteral)
564. {
565. $this->setPropertyValue($this->vocabulary->LABEL(),$resLiteral);
566. }

568. /**
569. * Set the value of the given property of this ontology resource to the given value.
570. * Maintains the invariant that there is at most one value of the property for a
571. * given resource, so existing property values are first removed.
572. * To add multiple properties, use addProperty.
573. *
574. * @param    object ResResource    $property
575. * @param    object ResResource    $value
576. * @access    public
577. */
578. function setPropertyValue($property, $value)
579. {
580. $this->removeAll($property);
581. $this->addProperty($property,$value);
582. }

584. /**
585. * Set the RDF type (ie the class) for this resource,
586. * replacing any existing rdf:type property. Any existing statements
587. * for the RDF type will first be removed.
588. *
589. * @param    object ResResource    $resResource
590. * @access    public
591. */
592. function setRDFType($resResource)
593. {
594. $this->setPropertyValue($this->vocabulary->TYPE(),$resResource);
595. }

597. /**
598. * Add a resource that is declared to provided additional information
599. * about the definition of this resource
600. *
601. * @param    object ResResource    $resResource
602. * @access    public
603. */
604. function setSeeAlso($resResource)
605. {
606. $this->setPropertyValue($this->vocabulary->SEE_ALSO(),$resResource);
607. }

609. /**
610. * Returns an array of ResResources that are recursively connected by $attribute
611. * in superProperty direction.
612. * If $onlyFindThisResResource is set to a ResResource, this function returns boolean
613. * if this distinct resource recursively is connected to the $startResource.
614. *
615. * @param    object ResResource    $startResource
616. * @param    object ResResource    $attribute
617. * @param    array                $attribute
618. * @param    object ResResource    $onlyFindThisResResource
619. * @return    array OR boolean
620. * @access    private
621. */
622. function _getSuperAttributeStatementsRec(& $startResource,& $attribute,& $attributeIndex, $onlyFindThisResResource = false)
623. {

625. $return = $startResource->listProperties($attribute);

627. if ($onlyFindThisResResource)
628. {
629. foreach ($return as $statement)
630. {
631. if ($onlyFindThisResResource->equals($statement->getObject()))
632. return true;
633. }
634. }

636. foreach ($return as $statement)
637. {
638. $attributeLabel=$statement->getLabelObject();
639. if (!in_array($attributeLabel,$attributeIndex))
640. {
641. $attributeIndex[]=$attributeLabel;
642. $subReturn = $this->_getSuperAttributeStatementsRec($statement->getObject(), $attribute, $attributeIndex, $onlyFindThisResResource);
643. }
644. }
645. if (isset($subReturn))
646. {
647. if ($subReturn === true)
648. return true;
649. return array_merge($return,$subReturn);
650. }

652. return $return;
653. }

655. /**
656. * Returns an array of ResResources that are recursively connected by $attribute
657. * in subProperty direction.
658. * If $onlyFindThisResResource is set to a ResResource, this function returns boolean
659. * if this distinct resource recursively is connected to the $startResource.
660. *
661. * @param    object ResResource    $startResource
662. * @param    object ResResource    $attribute
663. * @param    array                $attribute
664. * @param    object ResResource    $onlyFindThisResResource
665. * @return    array OR boolean
666. * @access    private
667. */
668. function _getSubAttributeStatementsRec(& $startResource,& $attribute,& $attributeIndex, $onlyFindThisResResource = false)
669. {

671. $return = $this->model->find(null,$attribute,$startResource);

673. if ($onlyFindThisResResource)
674. {
675. foreach ($return as $statement)
676. {
677. if ($onlyFindThisResResource->equals($statement->getSubject()))
678. return true;
679. }
680. }

682. foreach ($return as $statement)
683. {
684. $attributeLabel=$statement->getLabelSubject();
685. if (!in_array($attributeLabel,$attributeIndex))
686. {
687. $attributeIndex[]=$attributeLabel;
688. $subReturn = $this->_getSubAttributeStatementsRec($statement->getSubject(), $attribute, $attributeIndex, $onlyFindThisResResource);
689. }
690. }
691. if (isset($subReturn))
692. {
693. if ($subReturn === true)
694. return true;
695. return array_merge($return,$subReturn);
696. }

698. return $return;
699. }

701. /**
702. * Add a property to this resource.
703. * A statement with this resource as the subject, p as the predicate and o
704. * as the object is added to the model associated with this resource.
705. * If $this->rdfType is set, an additional statement about it's type
706. * is added.
707. *
708. * @param    ResResource                $property
709. * @param    ResResource/ResLiteral    $object
710. * @return    object ResResource
711. * @access    public
712. */
713. function addProperty($property,$object)
714. {
715. if ($this->rdfType !== false)
716. if (!$this->hasRDFType($this->rdfType))
717. $this->model->add(new Statement($this,$this->vocabulary->TYPE(),$this->rdfType));

719. return parent:: addProperty($property,$object);
720. }

722. /**
723. * Sets the rdf:type, that this distinct resource is instance of.
724. * If this value is set, the ontModel will add an additional
725. * statement about this resource and the fiven rdf:type
726. *
727. * @param object ResResource $resResource
728. * @access    public
729. */
730. function setInstanceRdfType($resResource)
731. {
732. $this->rdfType = $resResource;
733. }

735. /**
736. * returns the rdf:type, that this distinct resource is instance of.
737. *
738. * @return object ResResource $resResource
739. * @access    public
740. */
741. function getInstanceRdfType()
742. {
743. return $this->rdfType;
744. }
745. }
746. ?>
```

Documentation generated on Thu, 7 Jul 2005 13:42:32 +0200 by phpDocumentor 1.3.0RC3
